# A Micro Total Analysis System (μTAS) for the *In Situ*, Real-Time Tracking of Produced Water Discharges through Detection of PAHs and Other Aromatic Compounds

**DOI:** 10.1021/acs.est.4c08392

**Published:** 2024-11-28

**Authors:** Espen Eek, Christian Totland, Stephen Hayes, Bent Frode Buraas, Axel Walta, Ivar-Kristian Waarum, Erlend Leirset, Harald Lura, Rolf Christian Sundt, Arne Pettersen, Gerard Cornelissen

**Affiliations:** 1 Norwegian Geotechnical Institute, Sandakerveien 140, Oslo 0484, Norway; 2 Norsk Elektro Optikk AS, Østensjo̷veien 34, Oslo N-0667, Norway; 3 ConocoPhillips Skandinavia AS, Ekofiskvegen 35, Tananger 4056, Norway; 4 Equinor ASA, PB 8500, Stavanger 4035, Norway; 5 NMBU, Elizabeth Stephansens vei 15, Ås 1433, Norway

**Keywords:** PAH sensor, membrane extraction, fluorescence, produced water

## Abstract

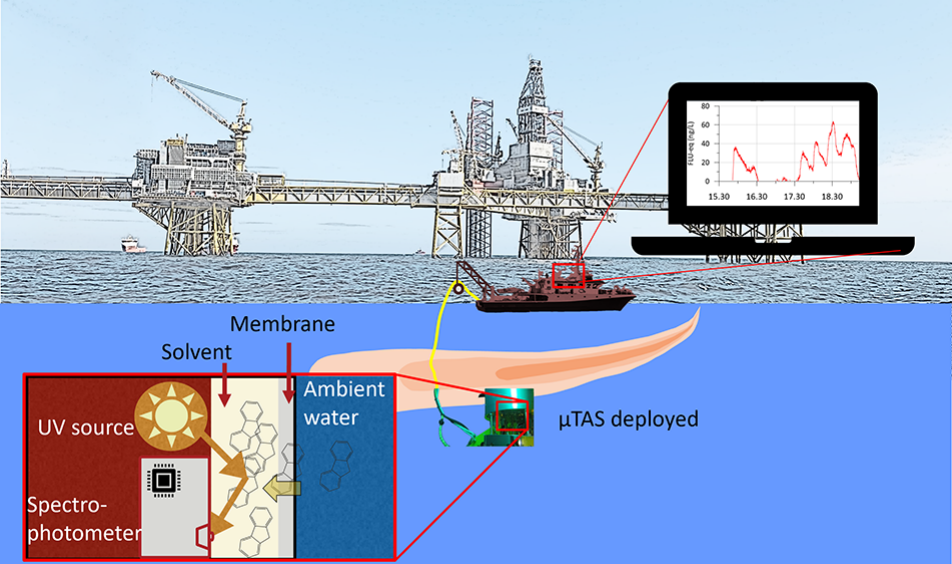

Here, we present a novel micro Total Analysis System (μTAS) for the measurement of poly cyclic aromatic hydrocarbon (PAH) and other aromatic hydrocarbons (AHs) in water at ng/L levels *in situ* and in real time (IMiRO). The μTAS is based on in-line membrane extraction followed by detection of extracted aromatic substances with fluorescence. An offshore field demonstration of the method was conducted close to produced water (PW) discharged in the North Sea. PW was monitored with the IMiRO μTAS and compared to results from a simultaneously conducted independent tracer release experiment, where fluorescein was added to the PW as a tracer. The μTAS monitoring and fluorescein tracer experiment showed similar ability to track the dispersion of the PW plume in space, depth, and time. Moreover, the method detected the sum of phenanthrenes and the sum of heavier PAHs with limits of detection down to 6 ng/L, with a response time of 6 min. The novel μTAS system opens up for *in situ* real-time discharge monitoring of both permitted and accidental oil or PW releases from oil platforms as well as other sources. Such monitoring can also be used to test and verify dispersion models used for environmental risk assessment.

## Abstract 2

Development and validation of a membrane extraction-based monitoring technology that can probe environmentally relevant concentrations of aromatic hydrocarbons in real time.

## Introduction

Ubiquitous use of oil products results in numerous planned and unplanned releases of hydrocarbons to water bodies. The offshore oil and gas production in the Norwegian Exclusive Economic Zone treat and discharge about 160 × 10^6^ m^3^ oil contaminated water (mostly produced water (PW)) and 1500–2000 t oil per year, mostly as residual oil via permitted PW discharges.^1^ Oil discharges reaching the water surface as free-phase oil can be detected as a visual sheen on the water surface or with IR or radar technology installed on surface production facilities. However, oil and numerous other associated hydrocarbons released with PW or smaller discharges will rapidly be mixed with ocean water and disperse and dissolve in the water column. This can result in plumes with relatively low concentrations of dissolved and finely dispersed hydrocarbons. PW consists of a mixture of a large number of aliphatic and aromatic hydrocarbons, some of them with functional groups (e.g., carboxylic, phenolic, or alcohol groups) that make them soluble or partially soluble in water.^2^ Several studies have shown that PW and particularly the polycyclic aromatic hydrocarbons (PAHs) and alkylphenols (APs) found in PW affect pelagic organisms 1–2 km away from the discharge.^3,4^ More subtle effects of PW discharges have been found as far as 10 km from the sources.^4^ Several recent studies have also found that semivolatile aromatic-like compounds (SALCs) are an important fraction of the oceanic dissolved carbon pool, originating from land-based, natural marine sources as well as from oil spills.^5−7^ Today, the fate and distribution of hydrocarbons in the water phase are typically monitored with active sampling, passive samplers, or with caged mussels.^8,9^ These methods have typical sampling and analysis times of weeks or months and thus will not capture short-lived accidental discharges or be suitable to map the variability in concentrations around the discharge as tidal current disperses the plume in different directions. Today, modeling based on discharge data, tidal currents, and other metocean data is the only tool to assess the distribution, fate, and effects of hydrocarbons from PW with a higher response time than months or years.^10,11^ Modeling requires knowledge of the time and rate of a discharge and is therefore also less suited to track unplanned discharges from leakages or accidents. Therefore, an obvious need exists for tools with the capability for real-time monitoring of hydrocarbons in water at concentrations that are relevant for environmental effects, often down to the ng per L range.^12,13^

Detection of any micropollutants (such as PAHs and AHs) in real time and *in situ* is notoriously challenging, as the concentration of the analyte is low and the concentrations of other potentially interfering substances in the matrix are much higher than the analyte concentration. Most attempts to design such sensor systems have relied on the interaction with a surface that is sensitive to the analyte or the interaction with light directly in the water phase (e.g., to measure fluorescence) for both the identification and quantification of the analyte in the same process. This strategy has major limitations in the ability to discriminate between analyte signal and signals from the vast variability of substances making up the matrix. In addition, when applying these strategies, the analytes are not separated from the matrix, which results in high detection limits in the high ppb or ppm range.^14^ Therefore, it is usually necessary to do the separation, volume reduction, detection, and quantification in multiple steps to achieve sufficient sensitivity and selectivity for quantification of microcontaminants. As a result, the monitoring of these microcontaminants usually relies on sampling followed by laboratory analysis.

PAHs and several other AHs emit fluorescence radiation when irradiated with UV-light. This property of aromatic compounds has been used as a part of many laboratory methods for analysis of PAHs,^15−19^ as well as in several commercially available sensors to detect PAHs or oil in water based on measurement of fluorescence directly in the water phase.^14,20−23^ However, these direct measurements of PAHs and other oil components suffer from two limitations. First, detection limits are high, typically several mg/L, which is 5–6 orders of magnitude above those encountered in the water column within a km from the PW discharge.^24^ Second, since the fluorescence is measured directly in the water phase without any prior separation of the analyte, other components may interfere with the fluorescence measurement, such as highly variable concentrations of suspended particles, marine snow, planktonic organisms, and dissolved organic carbon (DOC), all occurring at much higher concentrations (typically at μg/L to mg/L^25,26^) than the compounds of interest.

The micro Total Analysis System (μTAS) for the measurement of PAH and other AHs presented here is designed to solve these challenges. The novel μTAS sensor (“IMiRO”) is based on the principle of membrane extraction of hydrophobic compounds dissolved and dispersed in the water phase, followed by in-line (continuous) quantification of extracted aromatic compounds with fluorescence. By inclusion of the membrane extraction step, hydrophobic compounds (such as PAHs) are separated from potentially interfering compounds before quantification. Furthermore, the extraction upconcentrates analytes in the extraction phase where fluorescence is measured, strongly enhancing the sensitivity of the method. The principle exploited in the method developed here, using in-line membrane extraction, has been described in previous studies.^27−30^ However, it has not yet been applied in a field context, and no existing sensor has managed to measure PAH *in situ* and in real time with such sensitivity.

Based on this principle, a prototype of this novel technology was designed, constructed, and applied offshore. To independently test the validity of the measurement of AHs in the water phase done with the IMiRO μTAS, three different approaches were used: (1) laboratory calibration with PAH standards, (2) testing of measurement performance in the laboratory under field-relevant conditions (under variable pressure, temperature, and salinity), and (3) offshore field measurement of the fraction of PW in the water column. A significant and original element of the study was that the fractions of PW in the water column measured in the field with the IMiRO μTAS were compared with independently measured fractions of PW in a simultaneously conducted tracer release experiment (Sodium fluorescein tracer (CAS 518-47-8) added to the PW discharge). To our knowledge, this study is the first demonstration of *in situ* membrane extraction to measure microcontaminants including extensive field testing.

## Materials and Methods

### Design of the μTAS-PAH-Prototype

The μTAS PAH sensor described here was designed to do continuous membrane extraction of PAHs and other hydrophobic or organic compounds (HOCs) from water into a smaller volume of hydrophobic solvent, using a tubular silicone membrane, followed by the continuous quantification of extracted aromatic compounds with UV fluorescence.

**Figure 1:**
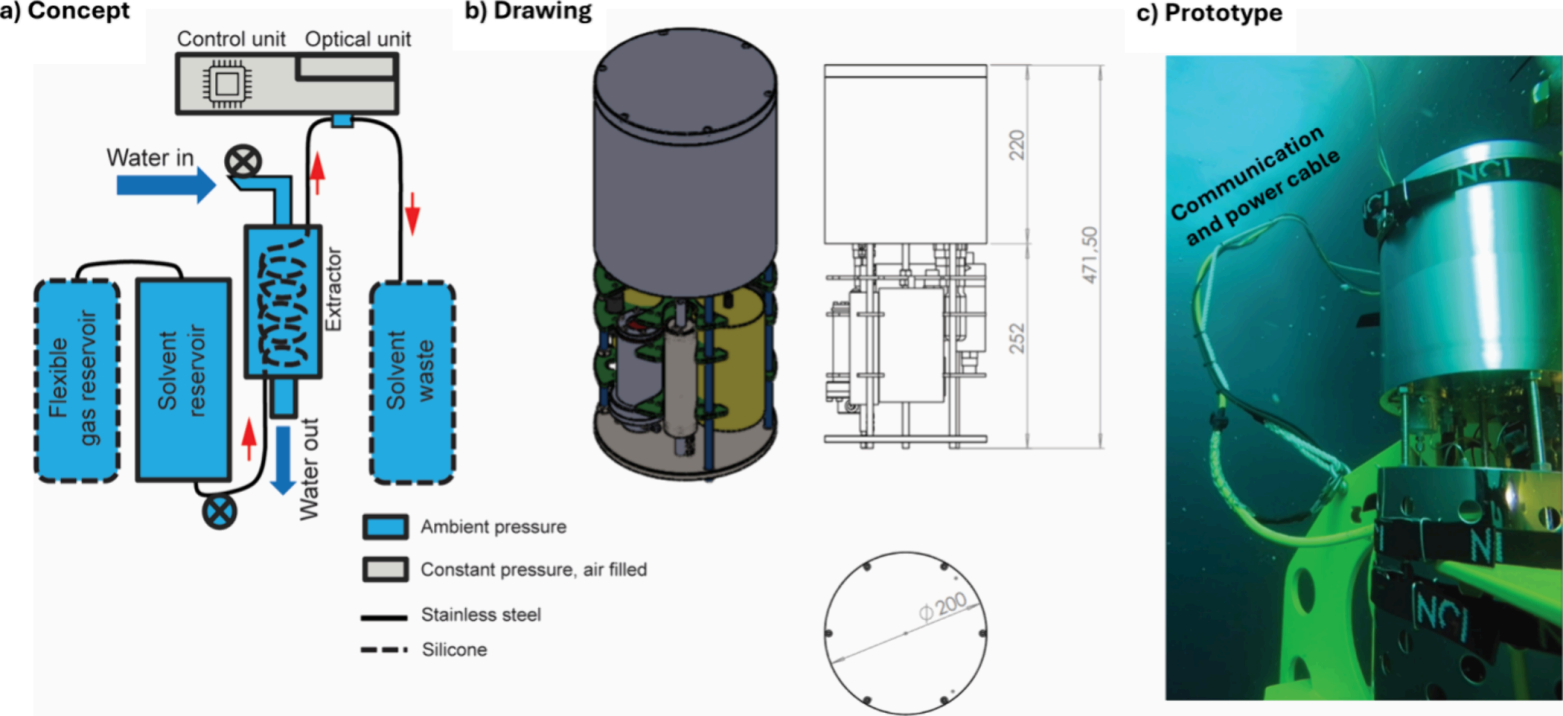
Outline of working principles of the IMiRO μTAS for AH-analysis at ng/L levels. (a) Schematic representation of the sensor principle. (b) Drawing of the assembled sensor. (c) Sensor submerged in water during offshore testing.

The automated analysis of AHs in IMiRO μTAS can be divided into two major steps: (1) Extraction of HOCs (including PAHs and other AHs) from the water through a 250 μm (wall thickness) tube silicone membrane into 1-hexanol solvent phase continuously pumped through the silicone tube. (2) Detection and quantification of extracted AHs with fluorescence (excitation at 255 nm, measuring the emission spectrum from 200 to 850 nm) as the solvent flows from the silicone tube through the flow cell (Figure 1a)).

The instrument outline and constructed prototype are shown in Figure 1, consisting of the following functional parts: (1) a water to solvent extractor, (2) an optical flow cell, (3) a UV-LED lamp (255 nm), (4) a Ocean optics FLAME-S-UV–vis-ES, 200–850 nm spectrometer (Winter Park USA), (5) Windows computer for system control and data communication, (6) a cable for data communication and power supply, and (7) a supply tank and waste tank for solvent. These parts and their functionality are described in detail below.

The extractor consisted of a flow-through chamber for the seawater that was made from a 108 mm long, iØ 31 mm stainless-steel tube, with a stainless-steel frame inside to support the solvent flow-through-membrane (800 mm long, iØ 0.5 mm AlteSil Silicone Tubing from Altec Ltd. with 250 μm wall thickness) in the water flow. A Sea bird scientific SBE 5 M Submersible Pump circulated the water constantly at a rate of 3 L/min through the extractor, continuously flushing ambient water over the outside of the silicone tube (the extraction membrane). Inside the tube, separated from the water by the membrane, 1-hexanol (99% from Acros organics) was constantly circulated in the opposite direction of the water flow, extracting hydrophobic compounds diffusing through the membrane from the water phase into the solvent phase.

A solvent was continuously pumped at 0.3 mL/min from a stainless-steel 500 mL reservoir with an HNPN micro annular gear pump through the silicone tube in the extractor and then into the flow cell (iØ 3.5 mm with volume 0.055 mL). In the flow cell, the solvent was irradiated with a LED-UV-lamp (255 nm) and the resulting fluorescence spectra were recorded with the FLAME-S spectrophotometer. Waste solvent flowed from the flow cell into a 500 mL waste container (in silicone plastic).

The solvent reservoir container, pipes, and other parts in contact with the solvent were made from stainless steel, except from the silicone tube in the extractor, 12 mm PTFE thread sealing band used on the pipe joint from the solvent reservoir and into the flow cell to avoid seawater from leaking into the solvent, and a sapphire glass window separating the solvent flow from the UV source and spectrometer. All fittings between pipes and containers were Swagelok stainless-steel fittings.

The optical system was located inside the pressure house at 1 atm, while the flow cell and solvent flow were outside the pressure house at ambient pressure. The excitation radiation and resulting fluorescent emission light from the solvent were exchanged between these compartments through the sapphire glass. A dichroic mirror combined with two filters was used to direct the excitation light from the LED to the flow cell while leading fluorescent emission light toward the spectrometer. Lenses were used to achieve near collimated light at the filters and dichroic mirror and focused light at the LED, flow cell, and spectrometer. A full fluorescence spectrum (200–850 nm) was recorded every second.

A microcomputer inside the pressure house ran software that controlled pumps for water and solvent flow, the spectrophotometer, and the UV source. An umbilical cable included twisted pairs for Ethernet communication, allowing extensive control of the hardware setup and data acquisition through the built-in computer. During deployment, recorded fluorescence spectra were transmitted to a topside computer, which ran a custom software that visualized a time series of selected fluorescence spectra in real time. The instrument was powered through the umbilical hose by a standard 24VDC power supply.

### Laboratory Testing

The IMiRO μTAS was tested in the laboratory to determine the sensitivity and selectivity of the method. Measuring PAHs with membrane extraction *in situ* at various depths means that the μTAS will experience environmental conditions different from those under which it was calibrated. The three factors temperature, salinity, and pressure can alter the chemical activity of the analytes and hence the transport across the membrane into the μTAS, resulting in variations in signal strength. To determine the dependence of the response on these parameters, calibrations were done (i) at 19.5 °C and at 3.1 °C and (ii) in deionized Direct-Q water and in water with 35 g of dissolved NaCl/L (Merck KGaA Darmstadt Germany) with PAH standards (mixture of PAH-16 Chiron AS). Furthermore, calibrations were done in (iii) an 840 L pressure test tank at pressures varying from ambient pressure (about 1 bar) to 11 bar, the total pressure at 100 m water depth (see Figures S3 and S4).

Fluorescence spectra of 1 μg/L fluorene (FLU), 1 μg/L phenanthrene (PHE), and a mixture of PAH-16 (1 μg/L of each component) dissolved in 1-hexanol and sodium fluorescein dissolved in water were recorded separately. This was done with an Agilent Cary Eclipse fluorescence spectrophotometer (Agilent Technologies, CA, United States).

Further experimental information is included in the Supporting Information document, including description of the calibration procedure, pressure tank testing, and procedure for determination of uncertainty and limit of detection (LOD).

### Field Testing

Offshore oil and gas producers in the Norwegian sector collectively carry out a water column monitoring survey every third year (WCMS). The IMiRO μTAS was included in the WCMS of 2021 to test the capability of this method to monitor and map the dispersion of the PW plume in the water column at the Ekofisk oil field in the North Sea. The WCMS 2021 took place from March 22nd to 28th, 2021 from the multipurpose vessel Esvagt Dee. The IMiRO μTAS was tested in parallel with *in situ* monitoring of a fluorescein tracer (added as a tracer to the PW discharge). The cruise was organized via a task force under Offshore Norge, an industry organization for companies with activities on the Norwegian continental shelf.

Approximately a total 13.7 Mm^3^/y PW is discharged as a part of normal operations from two discharge points, discharge point J (DP J) and discharge point M (DP M) at the Ekofisk oil production facility.^1^ The discharge volume is distributed 55%/45% between the two discharge points. The PW consists of formation water from the reservoir, return of injected seawater, as well as residuals of oil after separation of the crude oil and subsequent treatment of the water phase.

#### Offshore Sensor Deployment

The IMiRO μTAS prototype, a SAIV AS multi sensor with conductivity (salinity), temperature, and pressure (depth) sensors (CTD), as well as the Seapoint fluorescence sensor, were mounted on a 291 kg and 1084 × 1084 × 1100 mm stainless-steel sensor frame. The relatively large weight of the frame guaranteed depth stability during deployment in the water column. Through accurate and simultaneous recording of time, depth, and GPS location, *x*-*y*-*z*-*t* coordinates could be established for each single measurement. Figure 6 shows the track of the ship’s position during the PW-plume monitoring with IMiRO μTAS and the fluorescein sensor. Precruise modeling of the expected movement of the plume caused by changes in tidal currents at the time of the plume monitoring experiment (using existing models^10,11^) combined with real-time water current measurements from the oil field operator was used to plan the ship’s maneuvering to record transects along, as well as across, the dominant plume and depth profiles within the plume. Measurements were done at a 5–50 m water depth and at a 100–820 m distance from the PW discharge point.

### Produced Water Calibration and Response Time Correction

To determine the degree of PW dilution in the water column from the measured AHs with the μTAS, a calibration was done with dilution of a sample of the actual PW from DP M. The PW sample was collected before discharged to the water column the same day as the measurements were done in the sea (March 27th, 2021) and was immediately used to make a series of dilutions of this sample in seawater, with fraction of PW (v/v) ranging from 0.0001 to 0.0025. The water was mixed manually after each addition and continuously with the water pump in the μTAS.

#### Independent Determination of the Concentration of PAHs in the Water Column

The concentrations of PAHs and many other hydrocarbon compounds in samples taken of the PW discharge were analyzed annually by the operators of the oil production and reported to the authorities. The continuously measured fraction of PW in the water column (determined with μTAS) was multiplied with the concentration of individual PAHs measured in the PW to independently estimate the concentration of PAHs in the water column. These concentrations were compared with concentrations directly measured with IMiRO μTAS.

#### Tracer Experiment

To independently determine the fraction of PW in the water column, known amounts of fluorescein (sodium fluorescein, C_20_H_10_Na_2_O_5_, CAS Number: 518-47-8, Trade name MS-200, purchased from Schlumberger Norge AS, 150 g/L aqueous solution) were added to the PW at the discharge point DP M from 15:30–18:25 March 25th, 2021 and 06:30 to 12:15 March 27th, 2021 (UTC times) (with a permit from the Norwegian Environment Agency). The concentrations of fluorescein were measured in the water column outside the PW discharge with the Seapoint fluorescence sensor mounted, together with the μTAS sensor. Dilution factors were calculated from the ratio between the concentration of fluorescein in the PW before discharge and the concentration measured on the water column. The amount of fluorescein solution added and the amount of PW discharged per hour were recorded by the oil field operator and used to calculate the concentration of fluorescein in the discharged PW. This concentration varied between 0.75 and 1.4 mg/L in the undiluted PW during the fluorescein release experiment.

The background fluorescence in ambient water without the tracer was equivalent to 0.08 μg of tracer/L.

**Figure 2:**
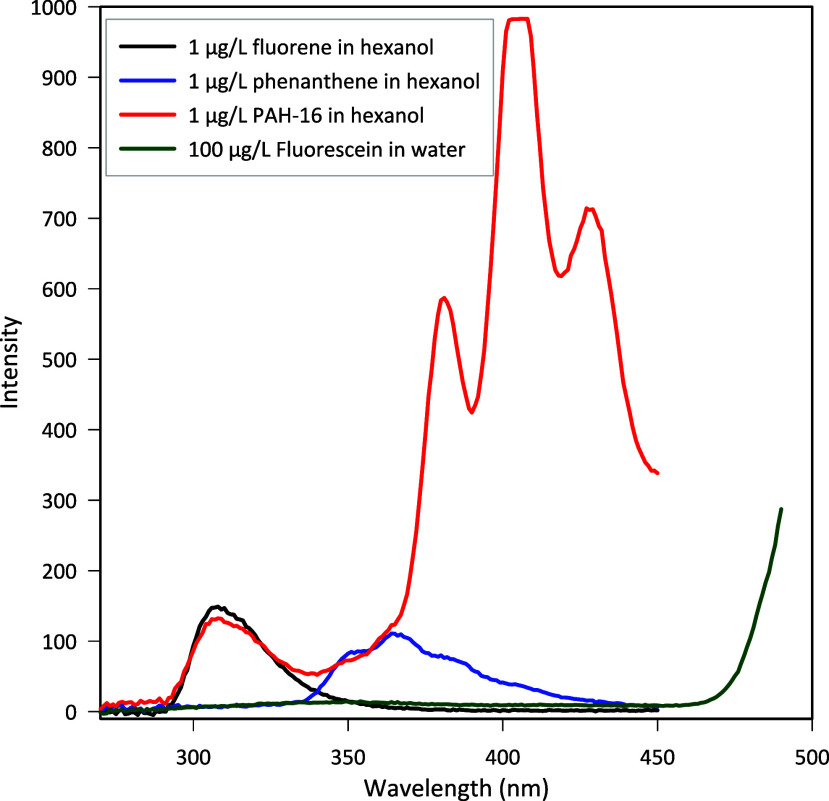
Emission spectra of FLU, PHE, and a mixture of PAH-16 at 1 μg/L of each PAH compound in 1-hexanol solution and 100 μg/L fluorescein in water. Recorded with excitation at 250 nm and corrected for 1-hexanol blank or water blank (fluorescein).

A concern with the simultaneous measurement of AHs (with the IMiRO μTAS) and the fluorescein tracer was that fluorescein could be extracted together with AHs and interfere with the quantification of the AHs in the μTAS. Figure 2 shows no overlap between the fluorescence (emission) spectrum of 100 μg/L fluorescein and the spectra of different PAHs (the analytes). Potential occurrence of such interference was also tested by running standards with sodium fluorescein concentrations of 4 and 20 μg/L, prior to the field measurements. The tests showed that the μTAS measurements were practically unaffected by varying concentrations of the water-soluble sodium fluorescein (see the Supporting Information).

### Data Treatment

Data from all sensors were reduced (averaged) from one data point per second to one per 10 s. The time of the measured concentrations with the IMiRO μTAS was corrected for the measured response time to enable a comparison with the near instant fluorescein measurements. AH-concentrations were determined from calibration with PAHs (see below) and adjusted to the temperature at the time of measurement, as measured by the CTD.

Figure S4 shows the baseline signal from the IMiRO μTAS measuring uncontaminated water in the pressure tank decreasing sharply during the first 30 min after starting up the instrument, before decreasing more slowly during the rest of the 5 h duration of the blank measurements. This background signal consists of the constant background fluorescence of hexanol and the signal from the decreasing amount of impurities extracted from the membrane and was corrected for by measuring a blank sample (DQ-water) at the beginning and at the end of the offshore measurements and in the lab, blanks were run after each sample.

Despite baseline corrections the fraction of PW was measured to be <0 in some areas. Although this coincided with areas where low PW fractions were expected, it also indicates that the baseline correction was not sufficient to fully capture the negative signal drift. This means that the measurements could slightly underestimate the fractions of PW and AHs.

## Results and Discussion

### Calibration with PAH Standards

Figure 2 shows fluorescence spectra for FLU, PHE, and a mixture of PAH-16 (1 μg/L in hexanol) and fluorescein (100 μg/L in water). The spectrum of the PAH-16 mixture between 300 and 365 nm was dominated by the fluorescence from the PHE and FLU as seen by the resemblance between the PAH-16, FLU, and PHE spectra in this area. The slightly lower intensity from 300 to 320 nm from the PAH-16 sample is probably due to the absorption of emitted light from FLU by the other PAHs in this sample. Above 365 nm, the heavier PAHs dominate the spectrum.

Naphthalene (NAP) fluorescence with emission between 300 and 400 nm (peaks at 321 and 333 nm) could influence the fluorescence signal at 303 and 360 nm, but NAP exhibits a lower quantum yield than FLU (Table S2) and also absorbs at a higher wavelength than used for excitation here and is therefore not expected to have great influence in the PAH-16 spectrum, where all PAHs have the same concentration.

**Figure 3:**
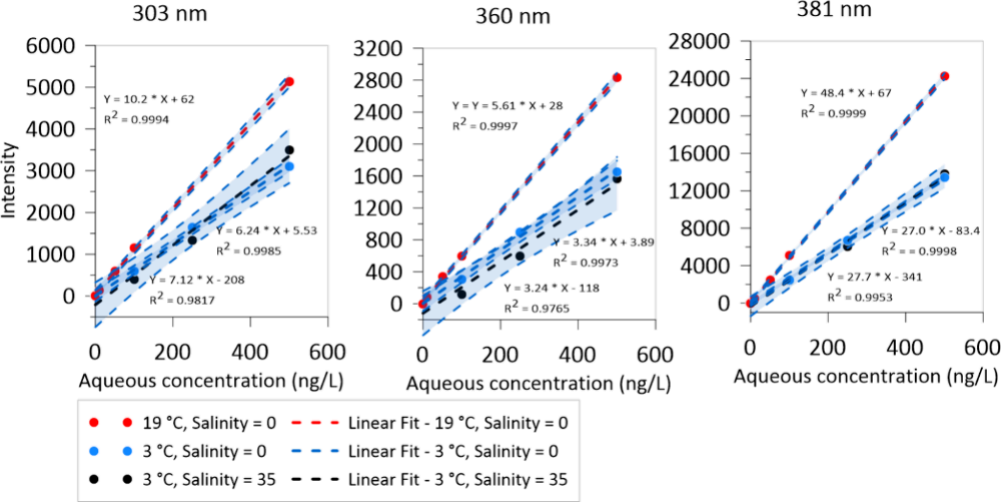
Calibration with PAH-16 standards (concentrations of individual compounds) at 19 and 3 °C in water with salinity 0 (deionized water) and salinity 35 (35 g NaCl/L). Blue dashed lines show linear fit and blue shaded areas show 95% confidence intervals for the linear regression of calibration data.

Based on the fluorescence spectra in Figure 2, the IMiRO μTAS was calibrated to measure three different PAH-equivalent concentrations: FLU-equivalents (FLU-eq) at 303 nm, PHE-equivalents (PHE-eq) at 360 nm, and higher ring PAHs at 381 nm (PAH-11-eq = anthracene (ANTH), fluoranthene (FLTH), pyrene (PYR), benzo[a]anthracene (B[a]A), chrysene (CHRY), benzo[b]fluoranthene (B[b]F), benzo[k]fluoranthene (B[k]F), benzo[a]pyrene (B[a]P), benzo[g,h,i]perylene (B[ghi]P), indeno[1,2,3-c,d]pyrene (IND), and dibenz[a,h]anthracene (D[ah]A)). The laboratory calibration (19 °C, atmospheric pressure, and in deionized water; Figure 3) showed that the method gives a good linear response (*R*^2^ > 0.999) from 0 to 500 ng PAH-16-mix/L.

### Effects of Temperature, Salinity, and Pressure on Sensor Performance

Calibration at 3 °C showed significantly lower sensitivity (by approximately 40%) at lower temperatures. This is believed to be caused by the lower diffusion rate through the membrane. No significant difference was found between the response in fresh water and that in salt water.

Measurements done in the pressure tank showed no effect of pressure changes between 1 and 11 bar (Figure S4) probably because ambient pressure always was maintained on both sides of the membrane (see Figure 1) and pressure changes therefore did not alter the chemical activity gradient across the membrane.

Thus, salinity and pressure were shown to have little effects on these measurements; however, temperature significantly affected μTAS sensitivity. Therefore, all concentrations measured with the μTAS were corrected for the difference between the sea temperature during measurements (as measured with the CTD) and calibration temperatures.

### Calibration of Fraction of Produced Water Diluted in Seawater

**Figure 4:**
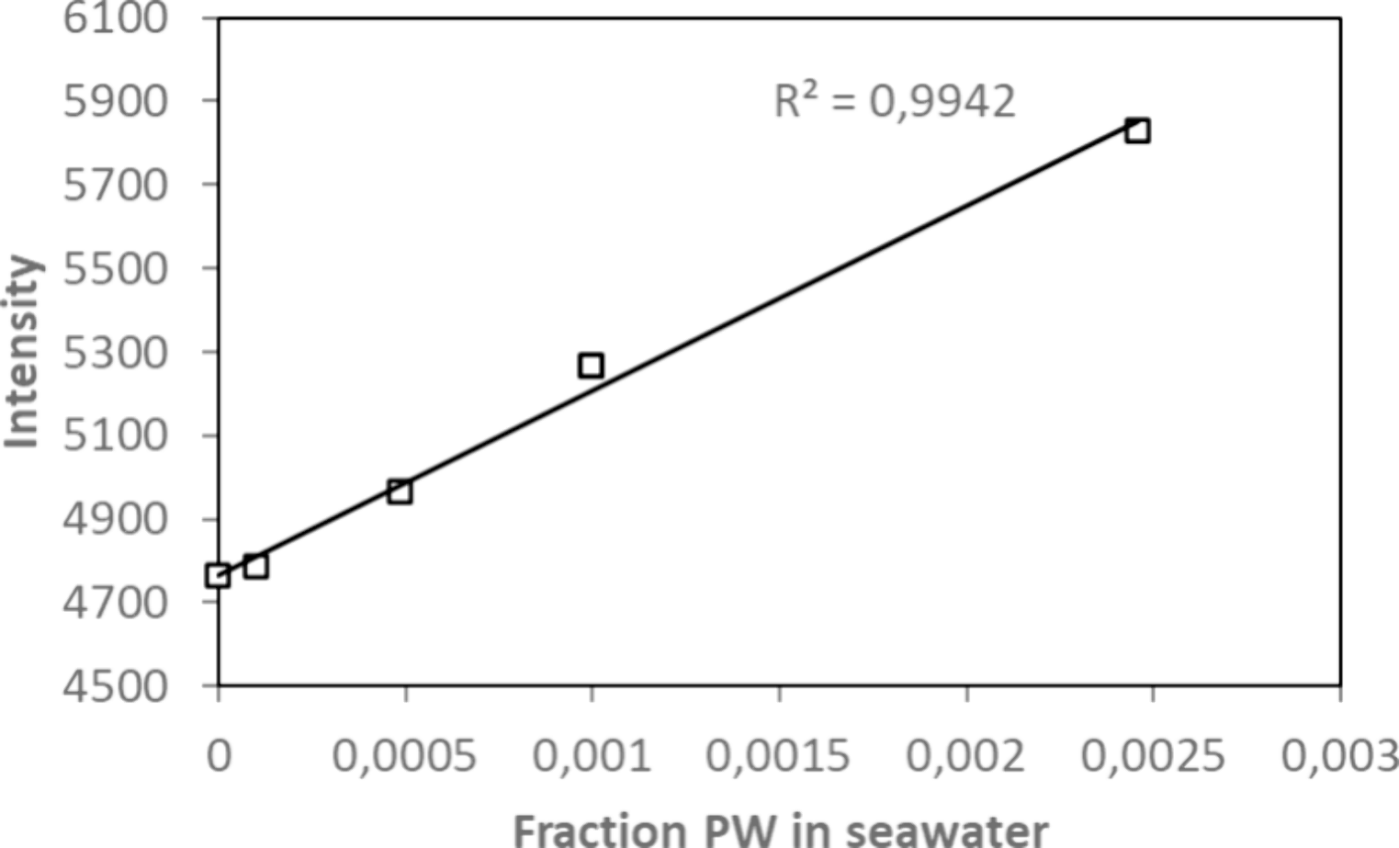
Calibration of the μTAS sensor at 303 nm with produced water obtained from the discharge point of DP M diluted in seawater. Calibrated with fraction of PW = 0.00246, 0.000997, 0.000486, and 0.000102.

Figure 4 shows the result of calibration of the IMiRO μTAS signal with an increasing fraction of PW mixed with seawater. The correlation was strong in the entire tested range of PW fractions (0.0001–0.0025). The calibration in Figure 4 was used to directly determine the fraction of PW in the water column with the IMiRO μTAS, using the AHs naturally occurring in PW as tracers. This measured fraction of PW was compared to the fraction of PW independently but simultaneously determined by measuring the fluorescein tracer from the tracer experiment. The comparison between these two tracers assumes conservative mixing of both tracers after discharge to the sea. Although different compounds in PW with different chemical properties (e.g., polar and nonpolar) will have different fate, it is likely that, at the μg/L to sub-μg/L concentrations of PAHs measured here, both the natural PAH tracers (nonpolar) and the artificial fluorescein tracer (polar) will be dissolved or finely dispersed in the water phase and that a large degree of conservative mixing can be assumed in the studied area (<1 km away from the source). Similar assumptions were made for the modeling of PW fate in the Nort Sea.^11^

The IMiRO μTAS was designed to minimize response time; nevertheless, extraction of PAHs and other aromatic compounds through the silicone membrane and the pumping of extraction fluid from the extractor to the flow cell imply that the signal will be delayed relative to changes in concentrations around the sensor. During the PW dilution calibration, the response time for the IMiRO μTAS was determined to be 370 s (Figure S6). This is similar to or lower than found in other membrane-based analytical systems like the membrane introduction mass spectrometer.^31^ The minimum achievable response time for the membrane extraction step in the μTAS is dependent on the internal diffusivity of each individual target compound in the silicon membrane. An internal diffusion model described for uptake in passive samplers^32^ allowed us to calculated the time to 90% equilibrium (*t*_90_) for a silicon sheet with the same thickness as the membrane of the μTAS (250 μm), using previously determined internal diffusivity coefficients in silicon^33,34^ from the same producer as used in our study. The calculated *t*_90_ for selected AHs and PAHs ranged from 90 s for NAP, to between 581 and 802 s for FLU, PHE, acenaphthene (ACE), acenaphthylene, and ANTH. Although the response time and time to equilibrium are not the exact same parameters (see the Supporting Information), the slightly higher estimates based on the diffusion model compared to the determined response time in the μTAS could be explained by lower retardation of the target compounds due to hexanol saturation of the μTAS membrane enhancing solubility and diffusivity.

### Produced Water Fraction in the Water Column

**Figure 5:**
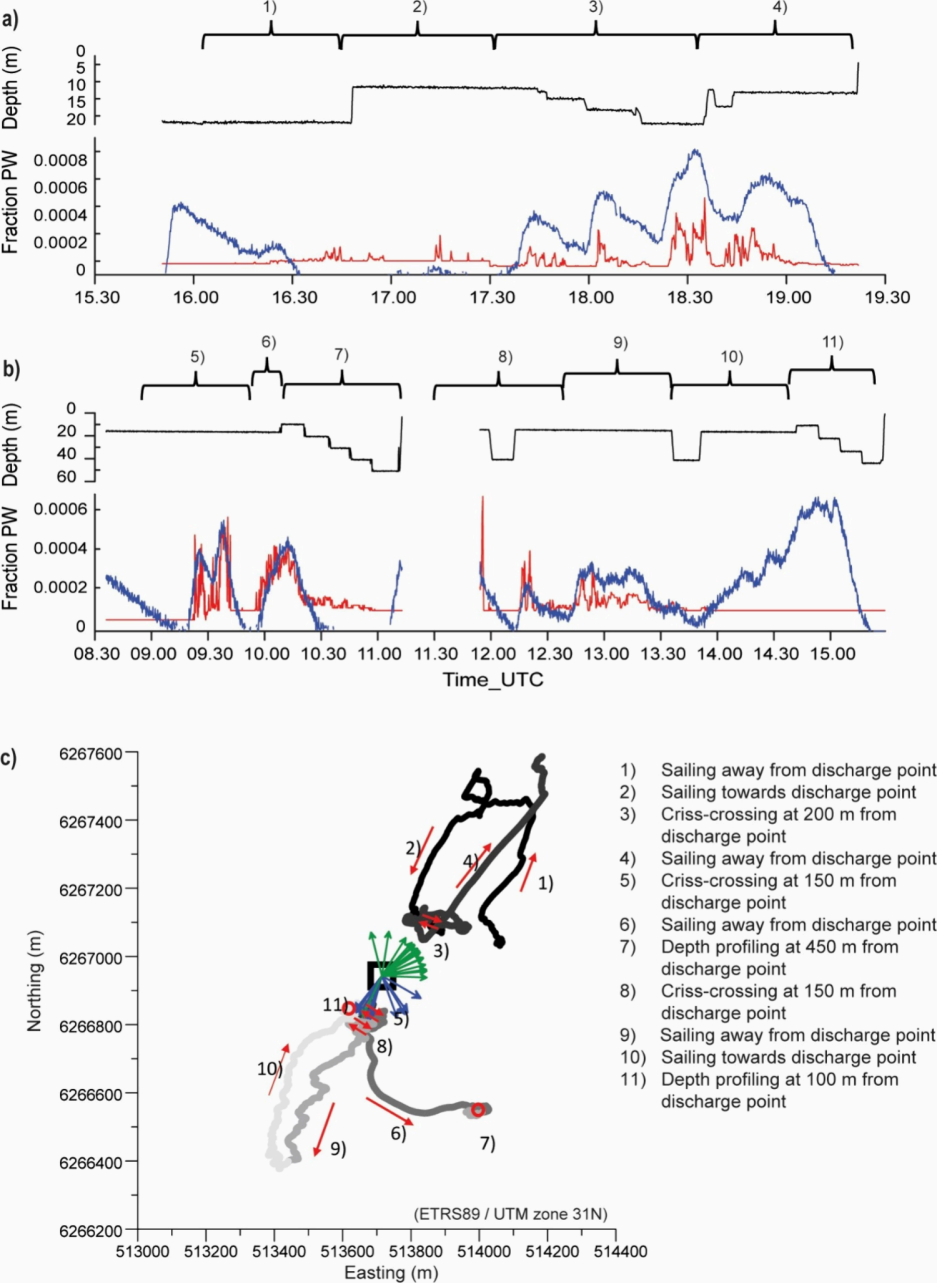
Panels (a) and (b) show the fraction of PW recorded with the IMiRO μTAS (Blue line) compared with the fraction of PW calculated with the sensor recording the fluorescein (red line) in the water measured in the monitoring campaign March 25th (a) and March 27th (b), 2021. The upper plots of panels (a) and (b) show sensor depth (black line). (c) Trajectory of where the ship moved. Black square shows the position of the DP M discharge point, green arrows show the current directions measured March 25th, and blue arrows show the current measured March 27th. Currents were measured by a current meter at the field center at 10 m depth 200 m south of the discharge point. Red arrows indicate the direction of movement by the ship carrying the sensors, and the red circles show the position where the depth profiles were measured. The numbering of these corresponds with the descriptions of the sailing direction in the figure and numbering of the corresponding plot sections above the plots in panels (a) and (b). Note that the fluorescein experiment (red lines) had ended during events 10 and 11.

Figure 5a,b shows the PW fraction measured both with the IMiRO μTAS and calculated from measured concentrations of the fluorescein tracer in the waters around the PW discharge. Figure 5c shows the maneuvering of the ship, and the black lines on Figure 5a,b show the depth of the sensors during the entire monitoring campaign.

The fraction of PW in the water column calculated from the fluorescein tracer varied from 0 to 0.0006, while the fraction measured with the IMiRO μTAS varied from 0 to 0.0009. The relatively low fraction of PW found in the water column indicates that the PW was rapidly diluted, more than 1000 times within 100 m of the discharge point. From Figure 5, it can be observed that the novel μTAS sensor and fluorescein tracer measurement detected PW peaks at the same time points. Thus, the two completely independent methods used to assess the PW fraction in the water column both measured an elevated fraction of PW in the same water masses when the sensors were close to the discharge and in the downstream current direction. Specifically, on March 27th, the fluorescein tracer release experiment ended at 12:15; subsequently, both the fluorescein sensor and IMiRO μTAS measured elevated but decreasing PW fractions in the water masses when moving away from the source (event 9 in Figure 5). The fluorescein tracer was then still in the water from discharge before 12:15, but its concentration was decreasing with the distance from the source. When the ship turned and moved toward the source again (event 10, Figure 5), the IMiRO μTAS measured increasing PW fractions toward the source, while the fluorescein sensor measured no fluorescein in the water, as the fluorescein release experiment had ended, and the PW released no longer contained fluorescein.

**Figure 6:**
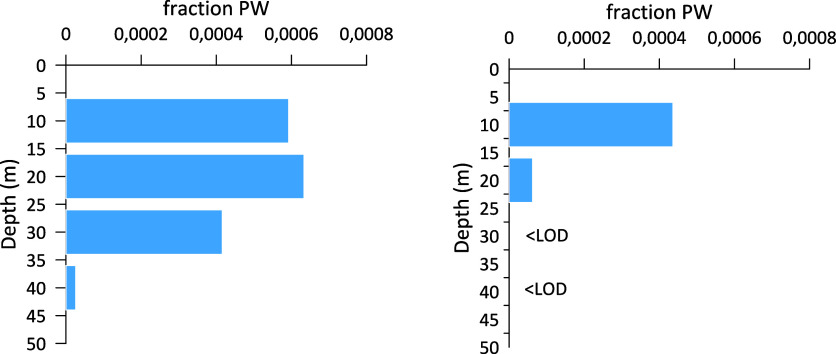
Profile of fraction of PW in the water column at two sites event 11: 140 m from DP M and event 7: 700 m from DP M determined with the IMiRO-uTAS sensor.

The IMiRO μTAS typically measured 1–2 times higher fractions of PW than did the fluorescein sensor. The sharper changes in PW fraction registered with the fluorescein sensor was attributed to its lower response time. At two locations on March 27th, a profile of the PW fraction was measured (events 7 and 11) in Figure 5. Both depth profiles showed that the PW plume was confined to the upper 30–40 m in the water column (Figure 6). At the point of release, the PW typically had a temperature around 70 °C. Temperature decreased rapidly when mixing in ambient water at 5.8–6.2 °C (measured with the CTD) but resulting in a lower density and buoyant PW plume. The distribution and dilution of the plume both horizontally and vertically agree well with modeled PW dilution in the water column around the discharge points.^35^

### *In Situ* PAH/FLU/PHE Concentration from μTAS Measurements

**Figure 7:**
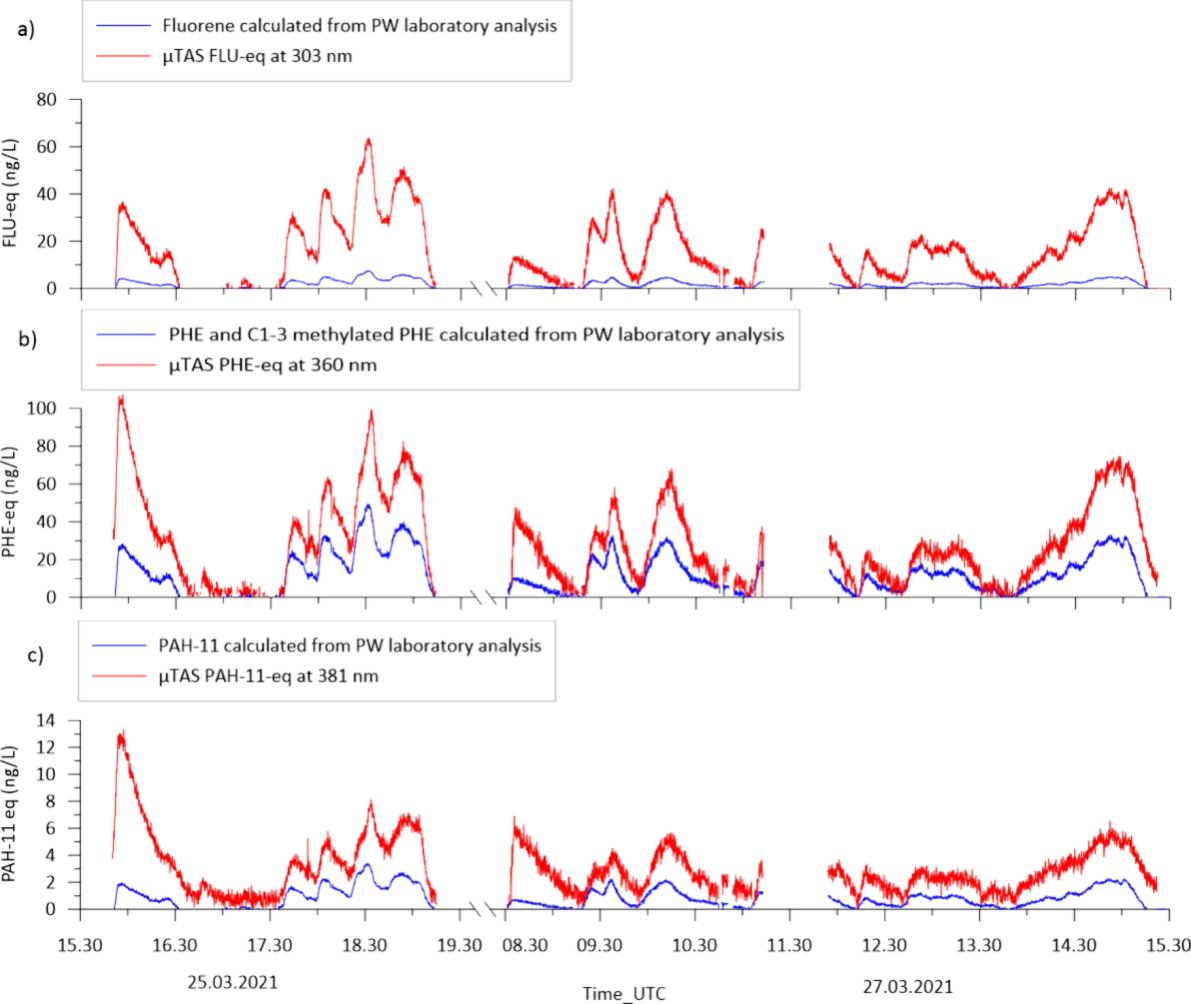
(a) FLU-equivalents measured at 303 nm and the concentration of FLU in the water column calculated from the FLU concentration in discharged PW and measured PW fraction, (b) PHE-equivalents measured at 360 nm and calculated PHE and C1–C3-methylated PHE concentrations, and (c) PAH-11-equivalents measured at 381 nm and calculated concentration of PAH-11 (ANTH, FLTH, PYR, B[a]A, CHRY, B[b]F, B[k]F, B[a]P, B[ghi]P, IND, and D[ah]A (4, 5, and 6-ring PAHs)).

After calibration of the IMiRO μTAS in the laboratory, it was used to continuously measure FLU-eq, PHE-eq, and PAH-11-eq in the water column *in situ* and in real time during an offshore expedition. Figure 7 shows these measurements with the μTAS compared with the concentrations of groups of individual PAHs independently calculated from measured concentrations in PW before discharge into the sea and the fraction of PW measured in the water column.

Both the measured fractions of PW and PAH-eqs showed that μTAS could clearly measure the plume outside the PW discharge. On the other hand, these measurements also showed that the μTAS quantification was not fully selective for the individual compounds. The measured FLU-eq overestimated the FLU concentration about 8.5 times (comparing measurements >1.9 ng/L (>25% of max)) indicating that several other components also contributed to the measured signal at 303 nm. Analysis of PW from DP M shows that the discharged PW contained concentrations of NAP, methylated NAP, phenols, benzene, toluene, ethylbenzene, and xylene that are 20–500 times the FLU concentration (Table S2). These one- and two-ring aromatic substances typically exhibit absorption (excitation) spectra covering 255 nm and emission spectra covering 303 nm^36,37^ and are also expected to be extracted together with the other AHs. Although their quantum yields are lower than those of FLU (Table S2), their high concentrations imply that they can be expected to contribute substantially to the measured fluorescence intensity (see the quantum yield multiplied by the concentration (Table S2)) explaining the higher concentration of FLU-eq measured with the μTAS than derived from PW concentrations (Figure 7a).

PHE-eq measured at 360 nm was compared with sum of concentrations of PHE and C_1_–C_3_-methylated PHE/ACE (*C*_PHE_). Both PHE-eq and PAH-11-eq measured with the μTAS agreed better with the concentrations derived from PW concentrations (overestimated by a factor 2.1 and 3.0, respectively) than the FLU-eq. This indicates less influence from other substances at the wavelength used for PHE-eq and PAH-11-eq. The level of deviation between concentrations determined *in situ* and concentrations derived from laboratory analysis, seen for PHE-eq and PAH-11-eq, could be explained by variations in PW composition or uncertainties in both methods. This shows that these two wavelengths were more selective toward specific groups of compounds than the signal at 303 nm.

The sum of FLU-eq PHE-eq and PAH-11-eq measured in the PW plume ranged from below 10 ng/L to about 170 ng/L. This is in the same order of magnitude as the concentrations of total PAHs found by deploying passive samplers at a 1 km distance from the discharge (25–350 ng/L).^24^ This underscores the accuracy of the μTAS in assessing PAH concentrations *in situ* and in real time down to the nanogram per L level.

### Reliability and Usefulness of the IMiRO μTAS

IMiRO μTAS is the first technology that managed the generation of real-time *in situ* data on aqueous PAHs at environmentally relevant concentrations (ng/L level) in ocean waters. Offshore field demonstration showed its ability to map a plume of PW discharged from an oil production platform. Measured offshore PAH concentration trends with depth, space, and time were validated by independent tracer measurements. In natural aquatic environments, the μTAS method, although still less accurate than water sampling and laboratory analysis, can provide significantly more detailed mapping in real time of the distribution of PAHs and other AHs than previously possible. This opens for more detailed studies of hydrocarbon discharges and field validation of discharge models widely used for environmental risk assessments in the offshore industry. This method could also be used to investigate discharges of PAHs and other SALCs with much higher temporal and spatial resolution than previously possible from point sources such as rivers, industry, and natural seeps.^5^ In addition, it could provide a useful tool for AH monitoring in many other anthropogenically impacted water bodies, such as wastewater and stormwater discharges. Also, sediment remediation or other construction projects in contaminated areas could benefit from this μTAS system, opening up possibilities to manage such projects based on real-time aqueous AH data, rather than grab sampling or passive sampling and laboratory analysis.^38^

The results presented here also demonstrated that the μTAS-based technology can be adapted for use in relatively harsh environments (tested in the laboratory to 100 m water pressure and in the field to 50 m in seawater) to solve monitoring tasks with microcontaminants close to background levels in large environmental systems, here represented by PW discharges into an open ocean system. This method takes advantage of current possibilities for miniaturization to enable the use of chemical manipulations for in-line separation of the analyte from the matrix in a manner similar to that in laboratory analysis and to replicate this in portable, real-time, *in situ* measurements.

## Supporting Information Available

Description of the following experiments: instrument calibration, pressure tank tests, fluorescein interference, sensor responsiveness, and fluorescing compounds in produced water (PDF)
