# Supplementary material for: A Micro Total Analysis System (μTAS) for the In Situ, Real-Time Tracking of Produced Water Discharges through Detection of PAHs and Other Aromatic Compounds
Source: Environ Sci Technol. 2024 Nov 28;58(49):21794–803. doi: 10.1021/acs.est.4c08392 (PMC11636241; doi:10.1021/acs.est.4c08392)
Supplement: Supplementary file 1 — es4c08392_si_001.pdf [file es4c08392_si_001.pdf]

Supporting information

A Micro Total Analysis System (μTAS) for the *In Situ*, Real-Time Tracking of Produced Water Discharges through Detection of PAHs and Other Aromatic Compounds

Espen Eek[1], Christian Totland[1], Stephen Hayes[1], Bent Frode Buraas[1], Axel Walta[1], Ivar-Kristian Waarum[1], Erlend Leirset[2], Harald Lura[3], Rolf Christian Sundt[4], Arne Pettersen[1], Gerard Cornelissen[1,5]*

*Corresponding author:

Espen Eek, email: espen.eek@ngi.no

Summary: 6 figures, 2 tables, 8 pages.

1 Calibration

When starting up the µTAS, for lab testing or measurements in the sea, the instrument was run in MQ-water for about 30 – 60 min (running 1-hexanol inside the membrane as under normal measurement) to clean the membrane. PAH-standards were prepared from dilutions of standard ampoules (Chiron AS) were used to calibrate the method and determine sensitivity of specific PAHs. Calibration of the µTAS were done by using the µTAS water pump to continuously circulate 1 L of the standard in the extractor.

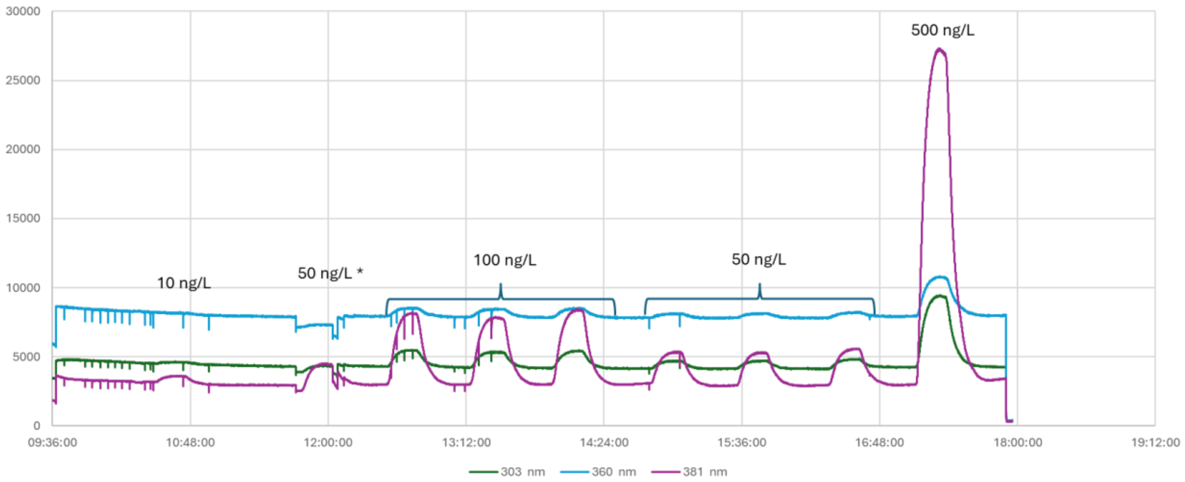

*Figure S1 Intensity measured during calibration at 19 C with PAH-16 standards (fig 3 in manuscript) *The second peak in the figure was not used in the calibration because of trouble with air bubble in the flow-cell.*

Figure S2 shows the calibration curves recorded at three different emission wavelengths before deployment in the field test.

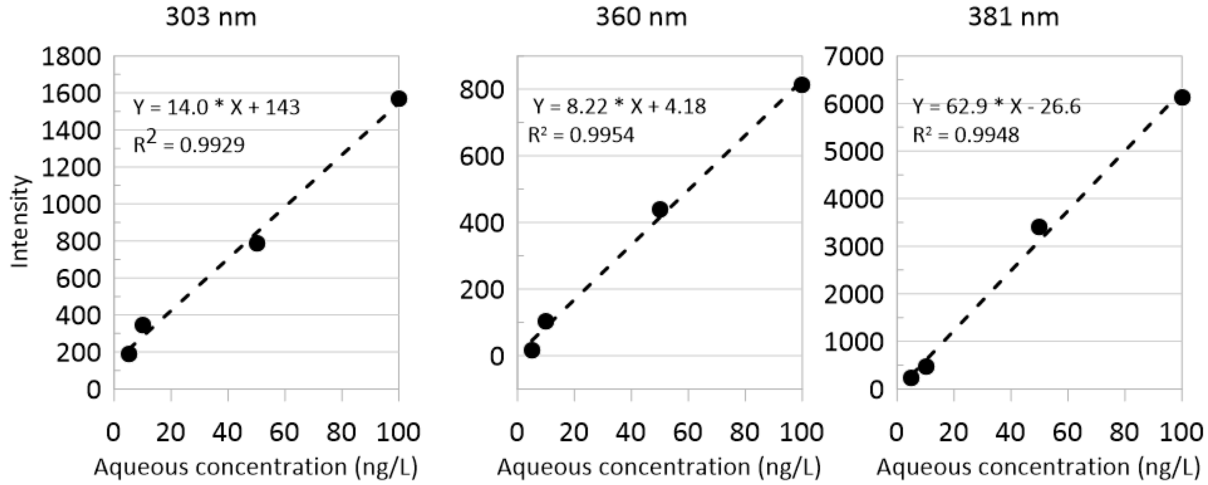

Figure S2 Calibration curves for standards with PAH-16 (mixture) in water measured with the µTAS, calibration of FLU-eq. at 303 nm, PHE-eq. at 360 nm and PAH-11-eq. at 381 nm.

1.1 Background signal and LOD

The instrument background signal (fluorescence measured in the µTAS instrument when exposed to clean water) consists of two major components: 1) a constant blank signal from hexanol, 2) signal from impurities extracted by the hexanol from the instrument (such as oligomers and impurities from the silicone tube). The hexanol signal was found to be constant when using analytical grade >99% 1-haxanol. The background signal from membrane impurities was found to be high after the instrument had been turned off and steadily decreasing during use, attributed to the leaching of impurities from the silicone tube when the tube is left with solvent inside without circulation (instrument off). The decreasing blank was corrected for by measuring blanks at the beginning and at the end of measurements when measuring in the sea, and between samples when operating the instrument in the laboratory.

To assess the signal variability in blank/low concentration samples, the standard deviation of signal recorded over five minutes in a blank sample (circulating Direct Q purified water) in the laboratory and from the average signal from four individual blank samples was calculated. The limit of detection (LOD) of PAHs was estimated as three times the standard deviation of these measurements. Three times the standard deviation of the signal from consecutive measurements of one blank sample was assumed to represent the minimum LOD for detection of increase in PAH concentrations above baseline in the water column during one measurement (FLU-eq.: 8 ng/L, PHE-eq.: 10 ng/L and PAH-11-eq: 2 ng/L). Three times the standard deviation between individual blank samples was used to calculate the realistic absolute LOD for PAHs for independent deployments of the instrument (FLU-eq.: 24 ng/L, PHE-eq.: 27 ng/L and PAH-11-eq: 6 ng/L)

1.2 Effect of pressure on the measurements

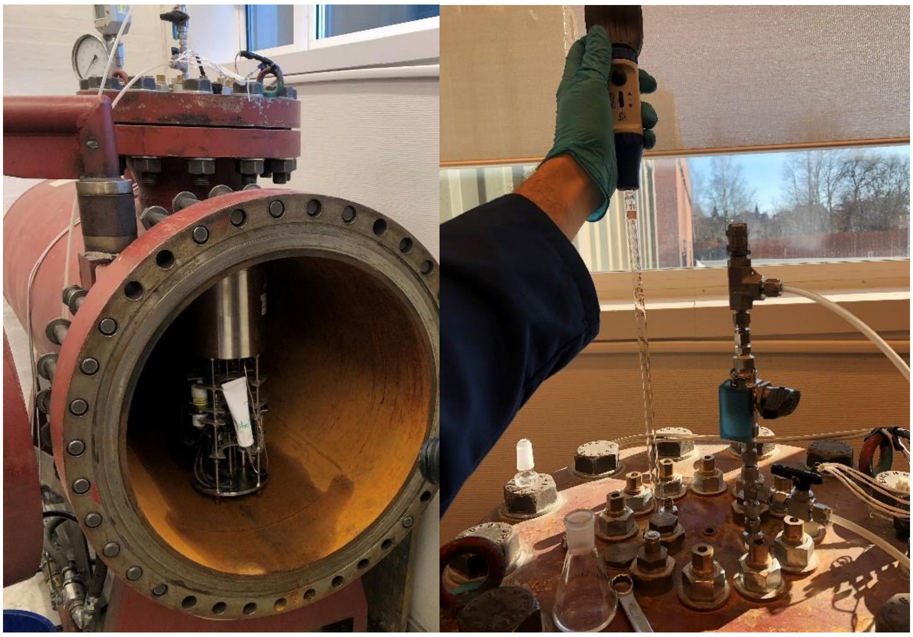

Figure S3 Testing of IMiRO-prototype in pressure tank

The ambient pressure will vary with depth during field operation of the µTAS. Since extraction is done over a non-rigid silicone membrane, the ambient pressure will by similar on both sides of the membrane. To see if these pressure changes could influence the measurements, the instrument was run in a water-filled pressure tank in the presence of single PAH-standards under pressures from 1 to 11 bar, covering water depths from 0 to 100 m.

The instrument was placed in an 840 L pressure tank filled with tap water (Figure S3). The instrument output was read through pressure tight wet connector from the instrument inside the pressure tank to an outside computer. For the first 4 hours background concentration readings in tap water was recorded as the pressure was gradually increased from atmospheric pressure to 4.9 bar, reduced to 2.7 bar again and raised to 11 bar.

After this test the pressure in the tank was released to atmospheric pressure again and single PAH/standards was added through an inlet in the top of the tank (Figure S3) before pressure again was raised. Standards was added to the tank to give the following concentration in the tank: Fluorene 118 ng/L, Fluorene 236 ng/L and Phenanthrene 118 ng/L.

The water in the tank was mixed using the water pump in the instrument (pumping rate 3 L/min) It was not expected that this would lead to complete mixing of the water in the pressure tank, and these measurements was not used to calibrate the instrument, only to test if the pressure would influence the measurements.

In addition to this test the pressure house (compartment with control units and optics) were tested to 50 bar (500 m) without leakage of water into the house.

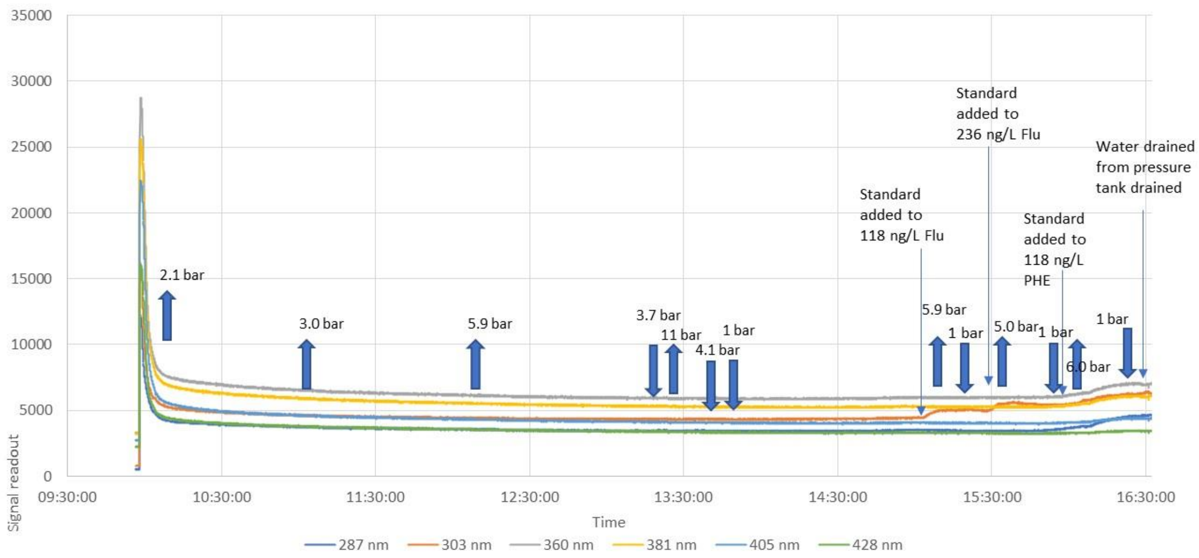

Figure S4 Measurement with IMiRO-prototype in pressure tank.

The test showed that the IMiRO-µTAS was running without technical problems and where able to extract PAHs from the water and maintain a stable signal in the pure tap water (background signal) under increasing and decreasing pressure ranging from 1 to 11 bar and measured consistently the increasing concentrations of PAHs added also under variable pressure (Figure S4).

1.3 Fluorescein interference

Blank samples with no PAHs, but with 4 µg/L and 20 µg/L concentration of Fluorescein (Sodium fluorescein tracer (CAS Number: 518-47-8)) was measured with the IMiRO-prototype, with a 27 min total exposure time to water with fluorescein.

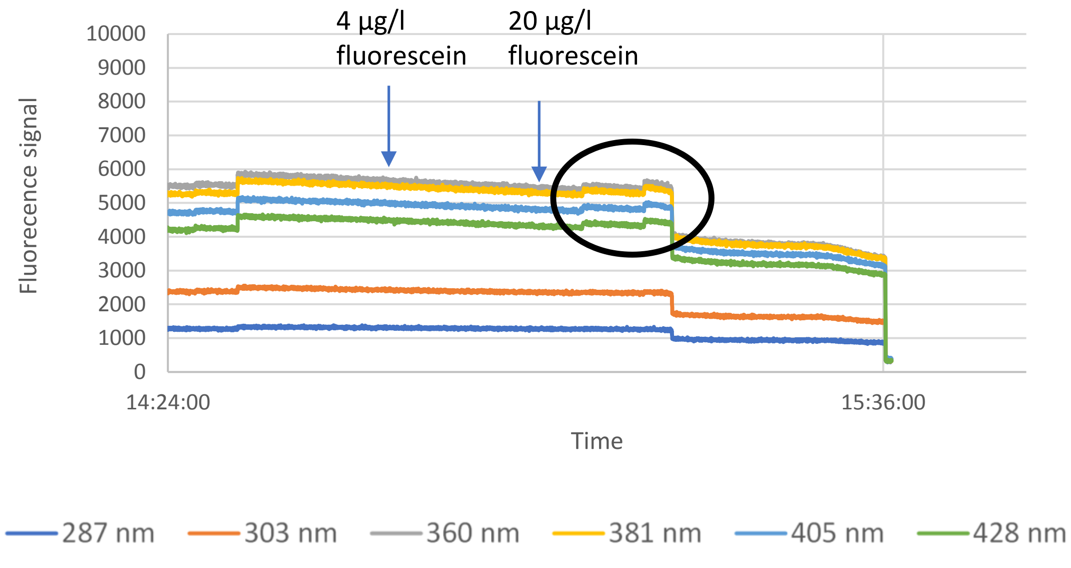

Figure S5 Measured signal with IMiRO-prototype during test with Fluorescein standard. Area with potential influence of Fluorescein in signals at wavelengths >360 nm. No interference from Fluorescein found at 303 nm.

Results from these measurements (Figure S5) show that there is no observable interference from the Fluorescein on the µ-TAS signal at 303 nm. At wavelengths from 360 nm and higher a weak increased signal can be seen after adding 20 µg/L fluorescein standard. However, even in 20 µg/L fluorescein this signal is weak. During tracer release experiment the maximum fluorescein concentration measured in the water column, with the independent fluorescein sensor, was 0.72 µg/L. This means that the potential interfering signal from fluorescein will not have any practical influence on interpretation of the data from the µ-TAS-sensor. This agrees well with the fact that fluorescein is a polar (ionic) organic compound and therefore will be poorly extractable in the extractor in the sensor and with the fact that the fluorescein excitation band (peak at 491 nm) and emission band (peak at 516 nm) are at good distance from the excitation wavelength (255 nm) and the emission lines (287 nm – 428 nm) used in the µ-TAS-sensor.

2 Responsiveness of the IMiRO µTAS

The time constant (T = time of 63.2% of maximum response) of the sensor was determined to be 370 seconds (6 min and 10 seconds) from the addition of PW to the measured response during the calibration for the produced water fraction of 0.001 (1:1000 dilution, Figure S6). The first 3 minutes after adding the PW, no signal was observed, due to the transport of solvent from the extractor into the flow-cell. The slow raise in signal after 3 minutes is interpreted as the effect of diffusion of the analytes through the membrane. This means that T of the membrane extraction is close to 3 mins, while T of the system as constructed is 6 min and 10 sec.

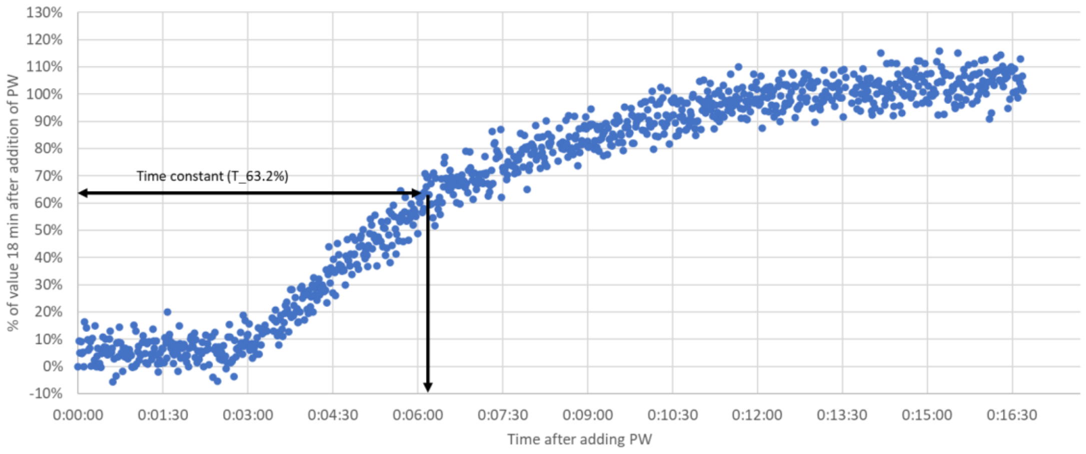

Figure S6 Responsiveness of IMiRO µTAS after addition of PW during the calibration of the sensor with PW at a 1:1000 dilution.

In order to estimate the variability in minimum achievable response time controlled by the diffusion time through the silicone membrane an internal diffusion model was used to determine the time to

90% equilibrium (t_{90})[1] in a silicone sheet with thickness 250 µm (equal to the membrane in the µTAS). Internal diffusivities[2, 3] determined for Altesil Silicone, also used in the µTAS. Table S1 lists the calculated t_{90}-values showing that these are in the same order of magnitude for the low to medium sized PAHs as the T (63.2% maximum signal) determined for the µTAS system and for the part of this attributed to trans membrane diffusion (3 min). This is explained by both the fact that this models predicts 90% equilibrium, compared to the response time determined at 63.2% of maximum signal and by lower retardation of the target compounds due to hexanol saturation of the µTAS membrane enhancing solubility and diffusivity. For the larger PAHs with slower internal diffusivity, t_{90} was also higher meaning that these compounds probably contribute less to the recorded signal.

Table S1. *Estimated time to 90% equilibrium based in internal diffusion in silicon sheets with 250 µm thickness for individual PAHs*

**Compound**	**log D (m2 s-1 at 20C) Altesil** Source [3] except Napthalenen[2]	**t_internal_90%_eq (sec.)**
Naphthalene	-9.23	90
Acenaphthene	-10.04	581
Acenaphthylene	-10.07	623
Fluorene	-10.06	609
Anthracene	-10.18	802
Phenanthrene	-10.18	802
Fluoranthene	-10.4	1331
Pyrene	-10.4	1331
Chrysene	-10.61	2159

3 Aromatic compounds in PW

Table S2 shows average concentrations of selected hydrocarbons in the discharged PW as analyzed and reported by the operators. Table 1 also shows the main excitation and emission bands for fluorescence from these compounds and estimated contribution to fluorescence intensity (concentration in PW multiplied with the quantum yield of the compound). Indicating that although fluorescence of BTEX compounds and naphthalene generally have lower quantum yield than for larger PAHs, the high concentration of these compounds means that they are still likely to contribute significantly to the fluorescence intensity in PW.

PAHs and other SALCs will also be present as a part of the background DOC in the North sea waters as in all world oceans originating from different sources[4, 5]. This will thus be part of the background when measuring the low concentrations in the sea or blanks using sea water. This concentration (< 1 ng/L for individual PAHs)[4] is however typically below the detection limits of this method.

__Table S2.__ Average concentrations of selected aromatic hydrocarbons in produced water discharged from DP J and M at Ekofisk , 2007 to 2021, analyzed by the operator. Spectral information about these compounds was taken from AAT Bioquest (https://www.aatbio.com/spectrum). Excitation wavelengths in __boldface__ are close to the excitation wavelength used in the instrument.

**Components**	**Reported concentration in PW 2020 – 2021 (N=3) Average ± std. dev (µg/L)**		**Exitation/ Emission band (nm)**	**Quant yield**	**Expected fluorescence contribution, (Quantum yield x concentration)**
	**DP J**	**DP M**			
Benzene	10500 ± 1900	5456 ± 900	**255**/278	0.07	4.09×10^{-1}
Toluene	3790 ± 860	4967 ± 450	**261**/284	0.17	8.56×10^{-1}
Ethylbenzene	80 ± 30	230 ± 22			
Xylene	660 ± 250	2000 ± 133	**265**/286	0.17	3.34×10^{-1}
Napthalene	120 ± 40	405 ± 70	311/322	0.23	8.53×10^{-2}
Acenaphthene	0.5 ± 0.3	1.4 ± 0.4	288/337	0.60	9.76×10^{-4}
Acenaphtylene	0.21 ± 0.17	0.005			
Fluorene	3.9 ± 1.5	10 ± 2	**261**/302	0.80	9.61×10^{-3}
Anthracene	0.03 ± 0.02	0.098 ± 0.02	356/397	0.36	4.63×10^{-5}
Phenanthrene and C1-C3 methylated PHE	6 ± 2.6	54 ± 6	**275**/365	0.13	2.29×10^{-3}
Fluoranthene	0.09 ± 0.07	0.13 ± 0.03	358/466	0.30	5.70×10^{-5}
Pyrene	0.26 ± 0.14	0.37 ± 0.05	336/384	0.32	1.46×10^{-4}
Chrysene	0.23 ± 0.12	0.24 ± 0.05	344/380	0.14	4.76×10^{-5}
C1-C5-methyl-phenols	2100 ± 380	8342 ± 102	**273**/300	0.08	1.83×10^{-1}
Sum methylated Dbenz-NAP-PHE-Anth	340 ± 140	950 ± 200			

*Dibenzo(a-h)anthracene, Benzo(g-h-i)perylene, Benzo(a)pyrene, Benzo(k)fluoranthene, Indeno(1-2-3-cd)pyrene, Benz(a)anthracene and Benzo(b)fluoranthene were not detected in the produced water at the discharge point.

4 References

(1) Liu, Y.; Xie, S.; Sun, Y.; Ma, L.; Lin, Z.; Grathwohl, P.; Lohmann, R. In-situ and ex-situ measurement of hydrophobic organic contaminants in soil air based on passive sampling: PAH exchange kinetics, non-equilibrium correction and comparison with traditional estimations. *J Hazard Mater* **2021**, *410*, 124646. DOI: 10.1016/j.jhazmat.2020.124646 From NLM PubMed-not-MEDLINE.
(2) Rusina, T. P.; Smedes, F.; Klanova, J.; Booij, K.; Holoubek, I. Polymer selection for passive sampling: a comparison of critical properties. *Chemosphere* **2007**, *68* (7), 1344-1351. DOI: 10.1016/j.chemosphere.2007.01.025 From NLM Medline.
(3) Rusina, T. P.; Smedes, F.; Klanova, J. Diffusion coefficients of polychlorinated biphenyls and polycyclic aromatic hydrocarbons in polydimethylsiloxane and low-density polyethylene polymers. *Journal of Applied Polymer Science* **2010**, *116* (3), 1803-1810. DOI: 10.1002/app.31704.
(4) González-Gaya, B.; Fernández-Pinos, M.-C.; Morales, L.; Méjanelle, L.; Abad, E.; Piña, B.; Duarte, C. M.; Jiménez, B.; Dachs, J. High atmosphere–ocean exchange of semivolatile aromatic hydrocarbons. *Nature Geoscience* **2016**, *9* (6), 438-442. DOI: 10.1038/ngeo2714.
(5) González-Gaya, B.; Martínez-Varela, A.; Vila-Costa, M.; Casal, P.; Cerro-Gálvez, E.; Berrojalbiz, N.; Lundin, D.; Vidal, M.; Mompeán, C.; Bode, A.; et al. Biodegradation as an important sink of aromatic hydrocarbons in the oceans. *Nature Geoscience* **2019**, *12* (2), 119-125. DOI: 10.1038/s41561-018-0285-3.
